# Evidence for a genetic basis of urogenital carcinoma in the wild California sea lion

**DOI:** 10.1098/rspb.2014.0240

**Published:** 2014-12-07

**Authors:** Helen M. Browning, Karina Acevedo-Whitehouse, Frances M. D. Gulland, Ailsa J. Hall, Jeanie Finlayson, Mark P. Dagleish, Karen J. Billington, Kathleen Colegrove, John A. Hammond

**Affiliations:** 1 Sea Mammal Research Unit, Scottish Oceans Institute, University of St Andrews, Fife, UK; 2 Unit for Basic and Applied Microbiology, Autonomous University of Querétaro, Querétaro, Mexico; 3 The Marine Mammal Center, Fort Cronkhite, Sausalito, CA, USA; 4 The Moredun Research Institute, Pentlands Science Park, Bush Loan, Penicuik, Midlothian, UK; 5 Pirbright Laboratory, The Pirbright Institute, Surrey, UK; 6 Zoological Pathology Program, College of Veterinary Medicine, University of Illinois at Urbana-Champaign, Maywood, IL, USA

**Keywords:** cancer, heparanase 2 gene, wildlife, odds ratio

## Abstract

Although neoplasia is a major cause of mortality in humans and domestic animals, it has rarely been described in wildlife species. One of the few examples is a highly prevalent urogenital carcinoma in California sea lions (CSLs). Although the aetiology of this carcinoma is clearly multifactorial, inbreeding depression, as estimated using levels of microsatellite multilocus heterozygosity, is identified as predictive for this neoplasia. On further analysis, this relationship appears to be largely driven by one marker, suggesting that a single locus might be associated with the occurrence of this disease in CSLs. In a case–control study, carcinoma was significantly associated with homozygosity at the Pv11 microsatellite locus. Pv11 was mapped to intron 9 of the heparanase 2 gene (*HPSE2*) locus, a very large gene encoding heparanase 2, which in humans is associated with multiple carcinomas. Correspondingly, immunohistochemical labelling in tissues was present in carcinoma cases within a single homozygous Pv11 genotype. To our knowledge, this is the first report of an individual locus being associated with cancer in any wildlife species. This adds emphasis to the study of *HPSE2* in other species, including humans and will guide future studies on this sentinel species that shares much of its diet and environment with humans

## 1. Introduction

While cancer is a major cause of mortality in domestic animals and humans, it remains rare in wildlife species. Some notable exceptions include devil facial tumour disease in the Tasmanian devil (*Sacrophilus harisii*) [1]; bandicoot papillomatosis and carcinomatosis syndrome in western barred bandicoots (*Perameles bougainville*) [2]; fibropapillomatosis in various species of sea turtle [3], a wide range of neoplasms in the Beluga whales (*Delphinapterus leucas*) from the St Lawrence Estuary in Canada [4,5] and urogenital carcinoma (UGC) in adult California sea lions (CSL) (*Zalophus californianus*) [6]. For a majority of these cancers the relationship with any causal agent, such as polycyclic aromatic hydrocarbon exposure in St Lawrence Beluga whales, remains only strongly associative.

Since the mid-1980s, UGC has been detected in CSL stranded along the central California coast [6] and is diagnosed in 26% of the adult stranded animals that are necropsied at The Marine Mammal Center, Sausalito, CA (between 1998 and 2012; F. Gulland 2012, personal communication). Recent studies have shown that a number of factors are associated with this UGC including an otarine γ-herpesvirus [7], infection with β-haemolytic streptococci [8] and persistent organic pollutants [9]. In addition, one study found that oestrogen receptor distribution was lower in intraepithelial lesions compared with normal genital epithelium and there were differences in p53 expression [10], suggesting that organic pollutants that interact with steroid hormone receptors and alterations in p53 may also play a role. There is also evidence for a genetic component in the aetiology of this cancer as particular major histocompatibility complex alleles have also been associated with an increased risk for occurrence of the carcinoma [11]. Furthermore, levels of multilocus heterozygosity (measured at unlinked microsatellites) predicted cancer in CSL, an effect attributed to inbreeding depression [12].

An independent study confirmed that the CSL dataset published by Acevedo-Whitehouse *et al*. [12] did indeed contain inbred individuals [13], a phenomenon most likely explained by the species' strong polygyny and philopatry [14], a result that offered support to the finding that relatively more inbred CSL are more likely to develop UGC. Further statistical analysis by Acevedo-Whitehouse (2005, unpublished data) found that the strength of this measure was driven by one microsatellite, Pv11 [15]. Pv11 has featured in many pinniped population genetic studies as it is a polymorphic locus in all three pinniped families [16–18].

Microsatellites have previously been used for detecting genetic associations to common diseases in humans and domestic animals [19]. The gene associated with human type 2 diabetes, *TCF7L2*, was identified by positional cloning through a genome-wide linkage scan of Icelandic families [20]. Several studies have found that a dinucleotide repeat microsatellite polymorphism, located in intron 55 of the fibrillin 3 gene, is significantly associated with the occurrence of polycystic ovary syndrome in humans [21–23]. The association between susceptibility to chronic pulmonary emphysema and microsatellite polymorphism in the haem oxygenase-1 gene promoter in humans has been well defined [24] as has the association of ZuBeCa3 microsatellite alleles and mammary tumours in various canine breeds [25]. While in some of these studies, the nature of the relationship between the microsatellite and candidate gene is not known, similar linkage studies and the use of microsatellites in case–control association studies, particularly in conjunction with the use of single nucleotide polymorphisms are becoming increasingly widespread, across many different taxa.

Here, we further investigate the association between microsatellite allele polymorphism and the occurrence of UGC in CSL. We discover the importance of a single microsatellite and a potential susceptibility locus for the development of UGC in this species.

## 2. Material and methods

Extended experimental protocols can be found in the electronic supplementary material.

### (a) Statistical analysis

Odds ratios were calculated using a binomial generalized linear model with a logit link function using the package R [26]. Likelihood ratio test *p*-values were reported. To investigate whether certain microsatellite alleles were associated with UGC, the median probabilities from a cumulative binomial distribution were calculated at each allele (comparing the number of UGC cases to the total number of animals genotyped as a specific allele). These were then compared across all alleles to determine the likelihood that UGC was more prevalent among animals with specific alleles.

### (b) Genotyping

Animals that died of UGC were diagnosed at necropsy. Control animals were classed as having died, been euthanized or released after treatment due to a condition other than typical UGC. Genomic DNA (gDNA) was extracted following either a proteinase K-chelex DNA isolation method followed by phenol chloroform purification or using the PUREGENE DNA isolation method according to the manufacturer's instructions. Amplification of three microsatellite markers (Pv11, M11a and Hg8.10) was undertaken via a multiplex polymerase chain reaction (PCR) using Qiagen Multiplex Master Mix (Qiagen) and fluorescently tagged primers (AB, Life Technologies). This enabled fragment analysis via automated capillary electrophoresis (ABI3700, Applied Biosystems in study A or Beckman Coulter CEQ 8000, UK in study B) and subsequent allele identification. To check for errors in the amplification, a minimum of 10% of the samples were run in triplicate and two negative controls were included in each plate.

### (c) Pv11 structure in skin and genital tissue DNA

Where possible, animals found to be homozygous at each of the five alleles identified in study B were selected. In total, 28 homozygotes were identified consisting of 18 allele 1, five allele 2, four allele 3 and one allele 4 animals, due to an absence of allele 5 homozygotes two heterozygotes were included. Skin gDNA was extracted as for genotyping; gDNA from lower genital tract tissue was extracted with the inclusion of an incubation step with alpha amylase (10% by volume) for 2 h at 37°C, prior to the addition of RNase A as per Buckles *et al.* [7]. A nested PCR protocol was used and PCR products of appropriate size (approx. 719 bp) were purified using MSB Spin PCRapace PCR purification kits (STRATEC Molecular, Germany). Where possible, 40 ng of DNA was submitted for sequencing. DNA sequencing was performed by DNA Sequencing & Services (MRCPPU, College of Life Sciences, University of Dundee, UK, www.dnaseq.co.uk) using Applied Biosystems Big-Dye v. 3.1 chemistry on an Applied Biosystems model 3730 automated capillary sequencer. The DNA sequences were analysed using the software program Geneious Pro v. 5.6.6 (Biomatters, available from http://www.geneious.com/).

### (d) Loss of heterozygosity

To investigate the occurrence of allele loss, 33 heterozygote animals from study B consisting of 26 controls and seven UGC positive animals were analysed. These animals were chosen as it was necessary to select heterozygote animals where both skin and corresponding lower genital tract tissue was available. gDNA extraction from the skin and lower genital tract tissues was as before. Amplification and analysis of the samples was undertaken as per genotyping with the modification that only the Pv11 microsatellite was amplified. To check for errors in the amplification, 50% of the samples were run twice and two negative controls were included. Allele loss was investigated using the formula (*N*_2_/*N*_1_)/(*T*_2_/*T*_1_), where *N* is the peak height of the assumed normal alleles (in this case, in the samples of gDNA from the skin) and *T* is the peak height of the potential abnormal alleles (in this case, the samples of gDNA from the lower genital tract tissue). Using this formula, allele loss is strongly suggested if the ratio is less than 0.5 or greater than 2.0 [27].

### (e) Location of Pv11 within heparanase 2 gene

A Southern blot was undertaken to identify the location of Pv11. For this gDNA from two species (harbour seal, *Phoca vitulina* and CSL) were used. These species are descended from a common canine ancestor and therefore conservation of the genetic structure is expected. gDNA was extracted as before. The Southern blot was undertaken with 5 µg of gDNA using the DIG system (Roche Diagnostics, Germany) as per the manufacturers guidelines and employed two independent probes, located either side of the microsatellite loci, with one including exon 9 of the *HPSE2* gene. Detection of the DIG-labelled probes was achieved by CSPD chemiluminescent detection via exposure to an X-ray film for 15 min. The film was then examined to identify the location of the hybridized probes.

### (f) Heparanase 2 gene transcription

Expression of *HPSE2* in tissues of the lower genital tract was investigated in six animals of different Pv11 genotype and disease state. RNA was extracted using Qiagen RNeasy extraction kit (Qiagen) and converted to complementary DNA (cDNA) using Invitrogen Superscript III Reverse transcriptase (Invitrogen). Integrity of the cDNA was confirmed by PCR using primers targeted to the mammalian β-actin gene. Two hemi-nested PCRs were carried out to amplify expressed *HPSE2* isoforms, initially to generate a small isoform fragment to confirm its presence followed by a reaction to generate the full-length isoform (approx. 1870 bp). Products from the full-length isoform PCR were resolved on a 1.2% agarose gel and post stained with 15 μl ethidium bromide prior to visualization of subsequent bands. The band patterns of the different animals were analysed and the two bands closest to the expected product size were gel extracted using a QIAquick gel extraction kit (Qiagen). Cloning of fragments was undertaken using pGEM-T easy vector (Promega) and protocol into TOP10 (Invitrogen) allowing the identification of positive transformants via blue/white screening. Plasmid purification of positive transformants was carried out using PureLink Quick Plasmid Miniprep Kit (Invitrogen) and sequenced using M13F and M13R primers. DNA sequence analysis was performed as before and identification of *HPSE2* isoforms present carried out using the online Basic Local Alignment Search Tool (http://blast.ncbi.nlm.nih.gov/Blast.cgi).

### (g) Heparanase 2 gene translation: immunohistochemistry

Formalin-fixed paraffin-embedded blocks containing tissues including lower genital tract tissues from 15 animals were supplied by Dr Kathleen Colegrove (University of Illinois). The samples included six UGC negative controls and nine animals suffering from UGC. The samples affected by neoplasia were graded according to their disease stage by Dr Kathleen Colegrove as previously described [10]. Two consecutive semi-serial sections (4 µm) were cut per tissue so as to allow an immunohistochemical methodology negative control for each sample, and mounted on Superfrost slides (Menzel-Gläser, Braunschweig, Germany). After blocking, the primary antibody (polyclonal, goat IgG raised against a peptide of human heparanase 2 (HPA2 (C-17), Santa Cruz Biotechnology, Inc., CA) was applied diluted 1 : 100 in 25% NRS/PBS at 4°C overnight. This antibody reportedly cross reacts with a range of species, including the dog. Moreover, the epitope is located at the C-terminal end of the protein which is highly conserved in all species. This region of the protein is highly conserved in CSL *HPSE2* and would be present in all the dominant HPA 2 isoforms based on the cDNA sequencing in this study. A negative control preparation for each of the tissue sections comprised substituting the primary antibody with normal goat serum at a dilution of 1 : 100. The slides were examined by light microscopy (Olympus BX50) and were scored ‘yes' if clear labelling was present and ‘no’ if labelling was absent. In ambiguous cases, findings were noted.

## 3. Results

### (a) Urogenital carcinoma in the California sea lion is significantly associated with homozygosity at a single microsatellite locus

Our previous work had indicated that CSL susceptibility to UGC was linked with inbreeding as assessed by microsatellite markers [12]. Two CSL sample sets collected consecutively were independently analysed to determine whether individual microsatellite loci were associated with the occurrence of UGC. Both datasets arose from the same source population of animals, but no animals were represented in both datasets. Control animals were considered as those that had died or were euthanized due to various reasons other than UGC and no control animals showed evidence of UGC on histopathologic assessment of tissues. Microsatellite genotypes of all the animals within each sample set were then independently determined and their relationship with the presence of UGC assessed.

**Figure 1.**
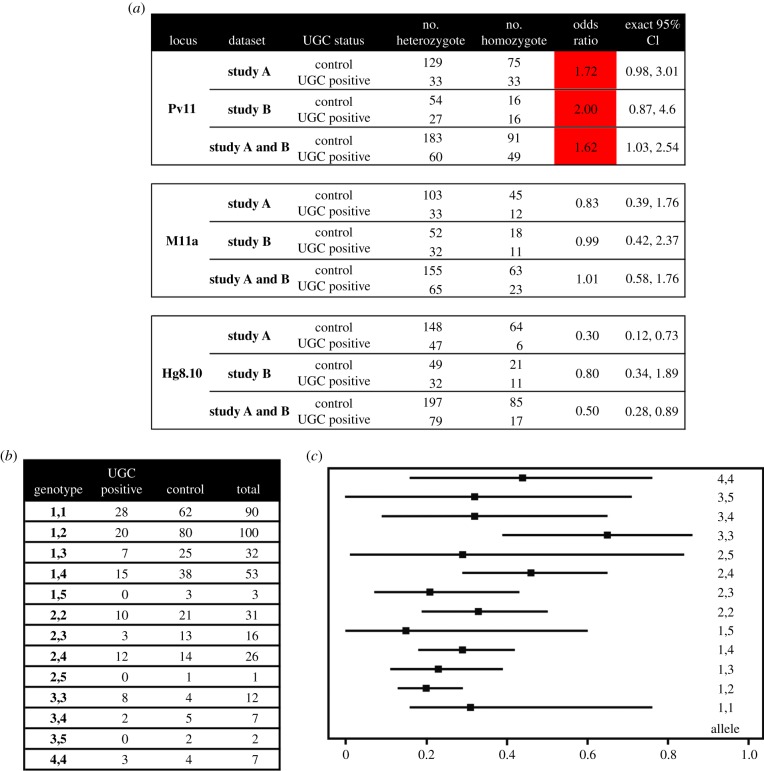
(*a*) The microsatellite allele diversity of UGC positive and control animals for each study alone and combined. Significant odds ratios are shaded. (*b*) Pv11 genotype distribution in CSL with and without UGC. (*c*) Median binomial probabilities for each allele combination and the 95% confidence limits (as the 2.5th and 97.5th percentiles from a distribution of probabilities generated from 0 to 0.99 for each allele). (Online version in colour.)

The first dataset (study A) included the animals from the initial study investigating the effect of internal (parental) relatedness on cause of death in CSL [12]. Eleven homozygous or heterozygous microsatellite genotypes were determined using capillary electrophoresis and homozygosity at one locus (Pv11), was found to contribute disproportionately to internal relatedness. This study additionally identified Pv11 as significantly associated with the occurrence of UGC (*p* = 0.05, figure 1*a*).

In the second dataset (study B), allele sizes and genotypes for Pv11 and two control microsatellites (M11a and Hg8.10) were assigned. In this dataset, although the odds ratio for the association between the occurrence of UGC and homozygosity at Pv11 was 2.0 (figure 1*a*), it was not statistically significant. However, when both datasets were combined, the association was statistically significant with an odds ratio of 1.62 (*p* = 0.04, figure 1*a*), indicating a link between genotype and the development of UGC.

### (b) Specific microsatellite allele combinations in California sea lion are not associated with urogenital carcinoma

To determine whether specific Pv11 genotypes were predictive of UGC, the datasets were combined (figure 1*b*). Two very rare alleles were only found in three animals from study A, with the remaining five being identical in both studies. To overcome the problem of bias due to the absence of some allele combinations in either group, a median probability and 95% confidence limits for each allele combination were generated from a binomial density function. This indicates the probability that an animal is a cancer case compared with a non-cancer case, given that particular genotype. The overlap between the lower confidence limit and the median probabilities was then compared between alleles. The median probability for genotype 3,3 was the highest (0.65) although its lower confidence limit overlapped with the median probabilities for 2,4 and 4,4 (figure 1*c*). A Cochran–Mantel–Haenszel *χ*^2^ test (CMH) was then used to investigate the association between UGC and homozygosity or heterozygosity across each allele group. There was a significant association between homozygosity and UGC (CMH estimate = 1.798, *p* = 0.006) but the odds ratio for allele 3 was approximately five-times higher than those for the other allele groups (odds ratio allele 1 = 1.57, odds ratio allele 2 = 1.47, odds ratio allele 3 = 7.5 and odds ratio allele 4 = 1.44). However, Tarone's test of homogeneity indicated that despite this variability, there was no evidence for a significant difference between these odds ratios (*p* = 0.163). This confirms the association between homozygosity at Pv11 and UGC (CMH with continuity correction = 7.09, *p* = 0.007, odds ratio 1.80, 95% CI 1.18, 2.73), but there is no support for any allele inferring greater risk.

### (c) Patterns of microsatellite diversity are not altered in California sea lion urogenital carcinoma

The Pv11 microsatellite was amplified from 60 gDNA samples taken from skin and lower genital tract from animals in study B to allow a comparison between healthy and diseased tissue from the same animals. Pv11 microsatellites comprised a variable region of CA dinucleotide units that were preceded by seven AC dinucleotide units. The end of the microsatellite had a mononucleotide C repeat sequence that also varied in number depending on the allele. However, allelic imbalance in the form of an apparent microsatellite contraction in the CA repeat was observed in only three animals; two allele 2 control animals and one allele 2 UGC animal (electronic supplementary material, table SI). This limited imbalance in diseased and control animals implies that instability is not an important factor in CSL UGC.

### (d) Urogenital carcinoma is not associated with the loss of Pv11 heterozygosity

Analysis of 33 Pv11 heterozygote animals from study B, identified allele loss in only one UGC animal (data not shown). Animal 9904(45) had a loss of heterozygosity ratio of 2.27, with ratios of less than 0.5 or more than 2 being strongly suggestive of allele loss [27]. However, the Pv11 genotype of this animal remained as 1,3, suggesting partial allele loss rather than complete allele loss. The spread of the loss of heterozygosity ratios calculated from the control animals ranged from 0.73 to 1.23 and the spread of ratios from the UGC animals ranged from 0.90 to 2.27. The results of a Fisher's exact test indicated that loss of heterozygosity is not significantly associated with the occurrence of UGC in CSL (*p* = 0.212).

### (e) Pv11 maps to the heparanase 2 gene locus in the California sea lion

The association of Pv11 homozygosity with UGC raises the possibility that this microsatellite may be linked to a larger genomic region in which genetic variation influences disease occurrence. Although no CSL genome is available, the dog genome assembly represents a canine species that shares a common ancestor with all the pinnipeds around 45 mya [28]. An identity search of dog genome scaffolds, using the CSL-specific flanking sequences around the Pv11 dinucleotide repeat, identified a highly identical region on dog chromosome 28 within intron 9 of the predicted *HPSE2*.

**Figure 2.**
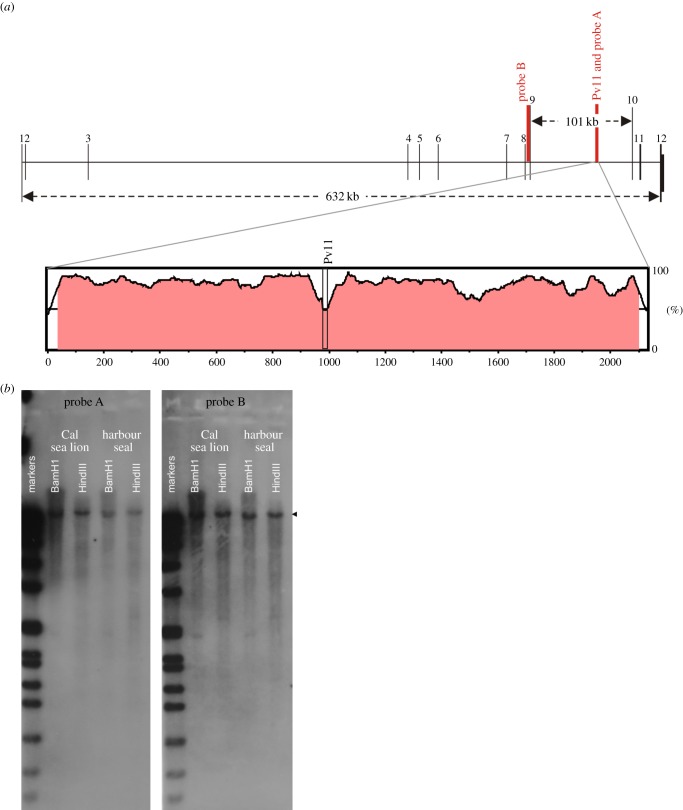
Microsatellite Pv11 is located within intron 9 of the CSL *HPSE2* locus. (*a*) Scale illustration of the *HPSE2* locus in the dog genome build with the exons numbered. The location of the probes used for Southern blotting and the Pv11 microsatellite sequence are marked and labelled in red. The pop-out window is a sliding window identity plot between 2.1 kb of CSL sequence over the Pv11 region compared with the same region in dog genome using 100 bp window length, red shading represents a minimum of 25% identity over 50 bp. (*b*) Pv11 and *HPSE2* probes hybridize to the same size DNA fragments in pinnipeds. Two Southern blots using the same digested DNA hybridized with two independent DNA probes.

To verify the location of Pv11 within *HPSE2* in the CSL genome, two approaches were taken. The first used comparative genomics to confirm that the exon structure of the *HPSE2* locus is conserved in mammals. The *HPSE2* gene has 12 exons and large intron sequences in all the genomes currently available including the dog, where the *HPSE2* locus is over 630 kb and intron 9 alone is 101 kb (figure 2*a*). This analysis facilitated primer design corresponding to conserved regions within intron 9 of the *HPSE2* locus. In the CSL, these primers amplified a 2.1 kb gDNA fragment that included the Pv11 sequence and was homologous to all the corresponding mammalian sequences (figure 2*a*). A sliding window analysis comparing the CSL intron 9 sequence with the dog genome confirms the orthology of these genes (figure 2*a*). The identity between these conserved non-coding sequences only drops below 50% over the unstable dinucleotide microsatellite region.

The second approach used the CSL intron sequence as a probe for Southern blotting. CSL gDNA was interrogated with two independent DNA probes, the first was the 5′ sequence flanking the Pv11 microsatellite (probe A), and the second was a CSL genomic sequence that we confirmed included exon 9 of the *HPSE2* locus (probe B) (figure 2*b*). In the dog genome, these two probe regions are separated by 69 kb. Both probes hybridized to the same CSL gDNA fragment (figure 2*b*). Taken together, these two lines of evidence confirm that Pv11 is within intron 9 of the CSL *HPSE2* locus.

The exon structure of *HPSE2* is conserved between mammals, although this large gene is known to produce numerous splice variants. To confirm the transcription and structure of *HPSE2* in the CSL, we characterized the cDNA sequences from the pancreas of eight animals using PCR primers located within the first and last exons. Several different primer pairs were used, with all producing a similar multiple banding pattern, indicating that a significant amount of splice variation and miss-splicing is likely occurring. As the previous genomic analysis predicted, the full-length transcript is highly similar to isoform 2 of the dog orthologue which contains all 12 exons, with a pairwise nucleotide identity of 97%. A predicted amino acid alignment comparing key mammalian species confirms the conserved nature of this gene and that the CSL product is most closely related to the dog and likely shares a very similar gene structure (electronic supplementary material, figure S1). Although there are several residues that are unique to the CSL sequence, these are either in areas of the protein that are more variable or conservative substitutions. The one notable difference is the histidine substitution for arginine at position 489 within the predicted heparin-binding motif (electronic supplementary material, figure S1). This substitution for a more positively charged residue may influence binding or reflect a difference in the heparan sulfate substrate in the CSL.

### (f) Heparanase 2 gene transcription and sequence diversity does not correlate with urogenital carcinoma

**Figure 3.**
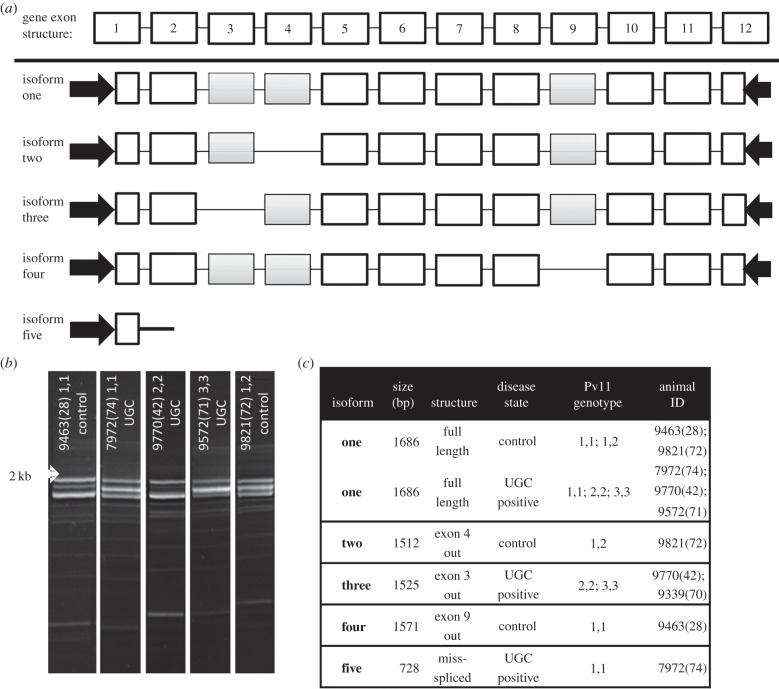
(*a*) Structure of the five identified CSL isoforms highlighting the variably spliced exons. Primer sites are indicated by the arrows. Isoform one was isolated from both control and UGC animals and is the full-length isoform containing all of the exons. (*b*) Gel electrophoresis of five of the *HPSE2* PCRs illustrating multiple banding patterns from animals of different disease states and genotypes. The expected product size of the full-length isoform is approximately 1870 bp, the arrow indicates the 2-kb marker. (*c*) Heparanase 2 isoforms identified with corresponding disease state in association with Pv11 genotype.

To determine whether differential transcription or splice variation was associated with UGC, the dominant *HPSE2* isoforms were amplified and cloned. The vast majority of the cloned PCR products were the full-length transcript, which corresponds to dog isoform 2, although as reported for other species several coding and non-coding splice variants were also identified (figure 3*a*). Two coding isoforms missing a single exon were the most common variants, isoform 2 is missing exon 4 and isoform 3 is missing exon 3. Overall, very little polymorphism was detected between individuals. These results confirm that *HPSE2* is a transcribed gene in the CSL that is structurally conserved when compared with closely and more distantly related mammalian species.

The same PCR amplification was performed using lower genital tract tissue in six animals from study B consisting of two control animals and four UGC positive animals of four different genotypes (figure 3*b*,*c*). In all six samples, the expected multiple banding pattern was visualized by gel electrophoresis with no correlation with disease state or genotype (figure 3*b*). The three dominant bands closest to the expected full-length transcript size were extracted for cloning and sequencing. Sequencing revealed the presence of the four isoforms produced via intron deletions previously identified and one novel truncated miss-spliced isoform (isoform 5) (figure 3*a*). However, the vast majority of the clones sequenced were the full-length CSL isoform 1 and there was no polymorphism between any of the products or animals.

### (g) Heparanase 2 protein is only present in cells in the genital tract from cancer-positive animals of one homozygous genotype

Although we did not observe any gross differences in *HPSE2* transcription patterns, we used a cross-reactive goat anti-human HPA2 polyclonal antibody to measure protein expression. A limited number of tissues (15) from study B were available that included four samples (9463(28), 7972(74), 9770(42), 9339(70)) that were included in the transcription analysis and were positive for mRNA expression.

**Figure 4.**
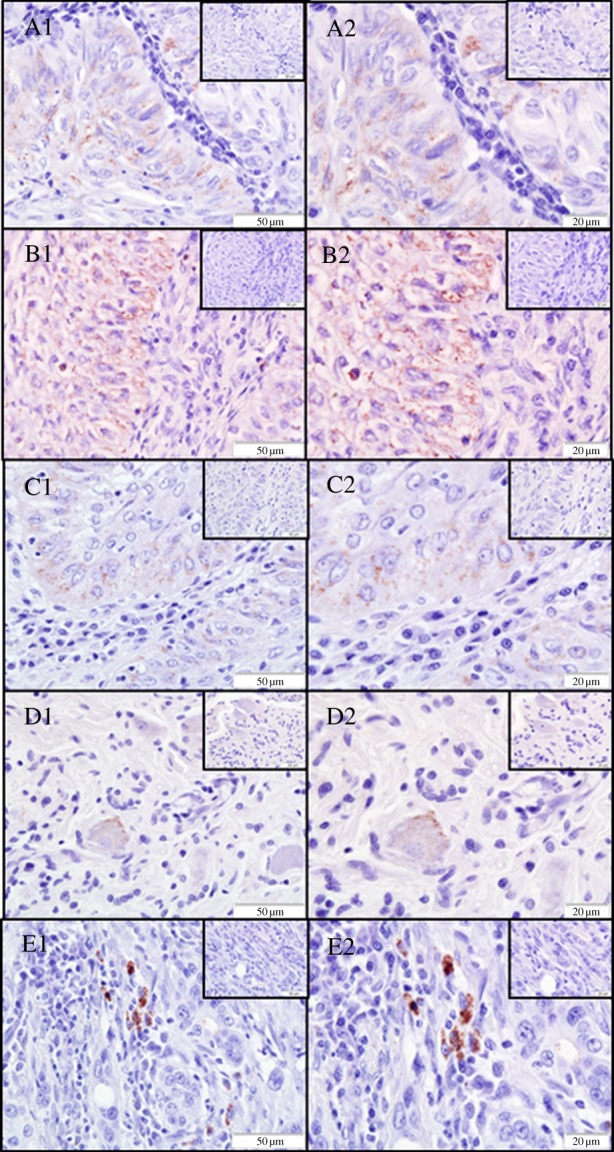
Examples of areas of positive immunolabelling (red pigment) of HPA2 identified shown at ×400 (A1, B1, C1, D1 and E1) and ×600 (A2, B2, C2, D2 and E2). All are tissues from female California sea lions of homozygous Pv11 genotype 1,1. Semi-serial negative control sections for each slide are shown inset. In animals 7972(74), 7997(68) and 9911(34), labelling was seen in neoplastic lower genital tract tissue. A1/A2: Animal 7972(74) (vagina): diffuse punctate to granular cytoplasmic labelling with variable amounts of the cytoplasm affected, ranging from none to 90%. Additionally, there was a large variability in intensity ranging from none to intense. B1/B2: Animal 7997(68) (cervix): predominantly granular and intense cytoplasmic labelling and mainly located in the periphery of the cells (presumed membrane associated). C1/C2: Animal 9911(34) (cervix): punctate to granular predominantly peri-nuclear labelling of the cytoplasm with variably 0–20% of the cytoplasm affected. Sections D1/D2 and E1/E2 illustrate the labelling pattern identified in animals 8431(69) and 9757(39) where cytoplasmic labelling was identified within neurons associated with the cervix (D1/D2) and in mononuclear inflammatory cells in the cervix submucosa (E1/E2), respectively. D1/D2: diffuse fine punctate to granular cytoplasmic labelling with the granular labelling more membrane associated. E1/E2: very intense granular labelling within the cytoplasm of a small number of mononuclear inflammatory cells and histiocytes with between 5 and 90% of the cytoplasm affected.

Of the 15 lower genital tract sections examined, three were positive for HPA2 labelling in neoplastic cells with diffuse punctate to granular cytoplasmic labelling in the basal layer of the epithelium. In a further two animals, fine punctate to granular labelling was present within the cytoplasm of neurons associated with the cervix and intense granular labelling within mononuclear inflammatory cells within the cervix submucosa (figure 4). There was no HPA2 expression in CSL urinary bladder or uterus despite the reports of high *HPSE2* mRNA expression in the equivalent human tissues [29]. Interestingly, all five sections where labelling was apparent were from Pv11 allele 1 homozygote animals with UGC (electronic supplementary material, table SII). There was no evidence of labelling in any of the other lower genital tract samples examined, including the UGC negative control Pv11 allele 1 homozygote control animals or the multiple negative control samples. Further studies with more animals are required to confirm and determine the nature of any association; however this study suggests that further to Pv11 homozygosity, expression of HPA2 in Pv11 allele 1 homozygous animals correlates with the presence of UGC. This implies that the *HPSE2* gene may have a role in the complex aetiology of this cancer.

## 4. Discussion

Here, we show that CSL homozygous at the microsatellite Pv11 are almost twice as likely to be carcinoma cases compared with those that are heterozygous. This association between Pv11 homozygosity and cancer may reflect inbreeding, resulting in reduced heterozygosity and therefore reduced fitness [30,31]. Homozygosity and reduced fitness has been noted in CSL before, where it was identified that more inbred individuals have longer recovery time from disease [12]. However with previous work suggesting that the relationship between inbreeding and cancer in the CSL was driven by the Pv11 microsatellite locus, there is an implication that this microsatellite holds greater importance in the development of the disease (Acevedo-Whitehouse 2005, unpublished data) and it is therefore possible that Pv11 is linked to a fitness-related gene [13,30,32].

The Pv11 microsatellite is located within intron 9 of the *HPSE2* gene, a very large locus in all mammals studied to date. *HPSE2* encodes heparanase 2 which has been implicated as a factor in multiple cancers in humans including UGC and is paralogous to heparanase (*HPSE*), a well-known oncogene. While we found no evidence that particular microsatellite alleles were associated with UGC prevalence, the expression of HPA2 was only found in tissues from animals suffering from UGC with a homozygous Pv11 allele 1 genotype. Although the precise role of this gene in the complex aetiology of this cancer remains enigmatic, our findings suggest the involvement of *HPSE2* and reveal differential genetic susceptibility to UGC within the CSL population.

Allele 1 was by far the most common Pv11 allele identified in this study, posing the question as to whether this genotype offers a survival advantage to this species. Yet, considering the restricted presence of HPA2, it was surprising that we found no significant association of this genotype, or indeed any other, with UGC in the dataset. Moreover, no differences were detected in the cDNA between any of the animals in any group or genotype. However, the number of animals studied, and the relatively low numbers of certain genotypes, although enough to confirm significance of Pv11 homozygosity, still requires significant expansion before more subtle trends can be confirmed. Additionally, UGC in CSL is likely to be caused by many factors. The odds ratios for the association between homozygosity at Pv11 reported here are the crude odds ratios which do not include the effect of other factors that have been previously linked to UGC in CSL (such as exposure to persistent organic pollutants and herpesvirus [7,9]). It is possible that the strength of the association may increase once these additional exposures have been accounted for.

There is a general and significant interest in the *HPSE2* gene in relation to carcinogenesis due to its sequence homology to the heparanase gene (*HPSE*) [29]. *HPSE* has been the subject of much research due to its involvement in neoplasia. *HPSE* encodes the enzyme HPA1, an endo-β-glucuronidase, which has the ability to break down heparan sulfate into smaller fragments [33]. It was initially believed that HPA2 had a similar action to HPA1. However, Levy-adam *et al.* [34] discovered that unlike HPA1, HPA2 does not exhibit enzymatic activity and also has stronger affinity to heparin and heparan sulfate than HPA1. They postulate that HPA2 may in fact work against HPA1, potentially inhibiting its action. Correspondingly, differences in HPA2 expression have correlated with several different human cancers [34–37]. In humans, *HPSE2* is located in a so-called loss of heterozygosity region in the genome (at 10q23–24) [29,38,39] that is predisposed to the loss of an allele rendering the locus homozygous. In cases where one allele has already undergone a mutation, as has been noted in familial retinoblastoma [39], the potential loss of the unaffected allele leaves the individual at risk of developing neoplasia.

In our study, it was striking that HPA2 labelling was restricted to animals suffering from UGC of a single homozygous genotype; however, labelling was not restricted to lower genital tract tissue. In two of the samples, the labelling was identified in neurons associated with cervix tissue and within mononuclear inflammatory cells in cervix submucosa rather than in cervix epithelium. Although there is scant information in the current literature regarding the presence of the HPA2 protein within tissues, there is a report of the increased presence of HPA2 in cells in the peripheral blood mononuclear cell fraction in humans with breast cancer [40]. Similarly, identification of HPA2 protein in neurons has also been previously reported in a study investigating the role of *HPSE2* in urofacial syndrome (OMIM#236730), a rare condition associated with dysuria and typified by unusual facial expressions [41]. There was a complete absence of labelling in lower genital tissues of other genotypes and disease state and additionally stage of cancer did not appear important as other samples of the same histological cancer grade, but genotypes other than 1,1 did not show labelling. The labelling pattern identified in our study offers further evidence to suggest that the Pv11 marker and the *HPSE2* gene are linked; however there is a clear need to extend the sample size as the examination of a larger number of samples, preferably alongside a standard tissue panel for each animal included, is required before definitive conclusions can be made.

## 5. Conclusion

To our knowledge, this is the first report of a single locus being associated with cancer in any wildlife species. Although *HPSE2* activity has not been fully characterized in any species, its antagonistic relationship with *HPSE*, itself an oncogene, makes it a highly likely protein to influence carcinoma. Indeed, the association between HPA2 expression with head and neck carcinoma in humans has been well established. The research presented here is part of a wider study that includes other potential causal factors including viral infection and contaminant exposure. While it may not be possible to mitigate against intrinsic factors such as homozygosity at Pv11, it may be possible to do so for the other interacting agents. And knowing what proportion of the population is homozygous at Pv11 could also assist in future prediction of mortality patterns and population trends, therefore informing on CSL conservation and management plans. Furthermore, this study emphasizes the role of *HPSE2* in mammalian carcinomas and may offer a model to help unravel the fundamental role of its protein.

## Supplementary Material

Supplementary Information
