# Supplementary material for: Evidence for a genetic basis of urogenital carcinoma in the wild California sea lion
Source: Proc Biol Sci. 2014 Dec 7;281(1796):20140240. doi: 10.1098/rspb.2014.0240 (PMC4213630; doi:10.1098/rspb.2014.0240)
Supplement: Supplementary Information [file rspb20140240supp1.pdf]

Supplementary information

Supplementary Results

IHC results – Other tissues

The tissue blocks obtained for the study incidentally contained tissues other than those from the lower genital tract. Due to the small number of studies that have identified expression of *HPSE2* at the protein level, these tissues were examined for labelling in addition to the main study. Of particular interest being tissues from the genital tract, other than cervix and vagina. The tissues examined included uterus, ovary, heart, diaphragm, stomach, urinary bladder and lymph node. Of these tissues translated protein has only been reported previously in the ovary in cases of benign and malignant ovarian cancer [1] and in the stomach in cases of gastric neoplasia [2]. Uterus and urinary bladder have been noted to show high levels of *HPSE2* mRNA [3, 4]. The findings are summed up in supplementary table II and the positive stomach sample and an example of a negative uterus sample are illustrated in supplementary figure 1.

Supplementary tables

Supplementary table I. Complete Pv11 sequencing results including those showing apparent allelic imbalance (indicated in bold). Allele five sequence was identified by ruling out the presence of the allele two sequence. XX: Sequence unavailable due to absence of tissue sample; X: inadequate sample quality

Animal No.	Pv11 genotype	UGC positive/Control	Skin DNA	Genital tissue DNA
**9184(14)**	1,1	Control	$(AC)_7G(CA)_{13}(C)_4$	$(AC)_7G(CA)_{13}(C)_4$
**9325(76)**	1,1	Control	$(AC)_7G(CA)_{13}(C)_4$	$(AC)_7G(CA)_{13}(C)_4$
**9114(2)**	1,1	Control	$(AC)_7G(CA)_{13}(C)_4$	$(AC)_7G(CA)_{13}(C)_4$
**9303(36)**	1,1	Control	X	$(AC)_7G(CA)_{13}(C)_4$
**9463(28)**	1,1	Control	$(AC)_7G(CA)_{13}(C)_4$	X
**9100(77)**	1,1	Control	$(AC)_7G(CA)_{13}(C)_4$	$(AC)_7G(CA)_{13}(C)_4$
**9196(78)**	1,1	Control	$(AC)_7G(CA)_{13}(C)_4$	$(AC)_7G(CA)_{13}(C)_4$
**8958(79)**	1,1	Control	$(AC)_7G(CA)_{13}(C)_4$	$(AC)_7G(CA)_{13}(C)_4$
**9008(80)**	1,1	Control	$(AC)_7G(CA)_{13}(C)_4$	$(AC)_7G(CA)_{13}(C)_4$
**9804(81)**	1,1	UGC positive	$(AC)_7G(CA)_{13}(C)_4$	$(AC)_7G(CA)_{13}(C)_4$
**7972(74)**	1,1	UGC positive	$(AC)_7G(CA)_{13}(C)_4$	$(AC)_7G(CA)_{13}(C)_4$
**7997(68)**	1,1	UGC positive	$(AC)_7G(CA)_{13}(C)_4$	$(AC)_7G(CA)_{13}(C)_4$
**8431(69)**	1,1	UGC positive	$(AC)_7G(CA)_{13}(C)_4$	$(AC)_7G(CA)_{13}(C)_4$
**9757(39)**	1,1	UGC positive	$(AC)_7G(CA)_{13}(C)_4$	$(AC)_7G(CA)_{13}(C)_4$
**9827(41)**	1,1	UGC positive	$(AC)_7G(CA)_{13}(C)_4$	$(AC)_7G(CA)_{13}(C)_4$
**9911(34)**	1,1	UGC positive	$(AC)_7G(CA)_{13}(C)_4$	$(AC)_7G(CA)_{13}(C)_4$
**7140(82)**	1,1	UGC positive	$(AC)_7G(CA)_{13}(C)_4$	$(AC)_7G(CA)_{13}(C)_4$
**7468(83)**	1,1	UGC positive	$(AC)_7G(CA)_{13}(C)_4$	$(AC)_7G(CA)_{13}(C)_4$
**6370**	1,1	UGC positive	$(AC)_7G(CA)_{13}(C)_4$	XX
**7819(75)**	2,2	Control	$(AC)_7G(CA)_{13}(C)_6$	$(AC)_7G(CA)_{13}(C)_6$
**9597(43)**	2,2	Control	$(AC)_7G(CA)_{13}(C)_6$	$(AC)_7G\mathbf{(CA)_{12}}(C)_6$
**7750**	2,2	Control	$(AC)_7G(CA)_{13}(C)_6$	XX
**8029(84)**	2,2	Control	$(AC)_7G(CA)_{13}(C)_6$	$(AC)_7G\mathbf{(CA)_{12}}(C)_6$
**9724(25)**	2,2	UGC positive	$(AC)_7G(CA)_{13}(C)_6$	$(AC)_7G(CA)_{13}(C)_6$
**9770(42)**	2,2	UGC positive	$(AC)_7G(CA)_{13}(C)_6$	$(AC)_7G\mathbf{(CA)_{12}}(C)_6$
**7131**	3,3	Control	$(AC)_7G(CA)_{15}(C)_4$	XX
**7867(73)**	3,3	UGC positive	$(AC)_7G(CA)_{15}(C)_4$	$(AC)_7G(CA)_{15}(C)_4$
**9339(70)**	3,3	UGC positive	$(AC)_7G(CA)_{15}(C)_4$	$(AC)_7G(CA)_{15}(C)_4$
**9572(71)**	3,3	UGC positive	$(AC)_7G(CA)_{15}(C)_4$	$(AC)_7G(CA)_{15}(C)_4$
**8059(30)**	3,3	UGC positive	$(AC)_7G(CA)_{15}(C)_4$	$(AC)_7G(CA)_{15}(C)_4$
**7159**	4,4	Control	$(AC)_7G(CA)_{16}(C)_4$	XX
**8921(33)**	4,4	UGC positive	$(AC)_7G(CA)_{16}(C)_4$	X
**9254(3)**	2,5	Control	$(AC)_7G(CA)_{17}(C)_3$	X
**9871(23)**	3,5	Control	X	X

Supplementary table II. Results of HPA2 immunolabelling of lower genital tract tissues of various Pv11 genotype and disease states. LGIL: Low grade intraepithelial lesion; HGIL: High grade intraepithelial lesion; IC: Invasive carcinoma. Numbers in bold indicate the samples where labelling was within neurons associated with the cervix tissue (8431(69)) and in inflammatory cells in the cervix tissue (9757(39)).

Pv11 genotype	UGC positive/control	animal ID	genital tissue labelling	genital tissue (lesion grade)
1,1	Control	9184(14)	Negative	Cervix
		9325(76)	Negative	Cervix +vagina
		9114(2)	Negative	Cervix+vagina
		9463(28)	Negative	Cervix
	UGC positive	7972(74)	Positive	Vagina (HGIL)
		7997(68)	Positive	Cervix (IC)
		**8431(69)**	Positive	Cervix (HGIL)
		**9757(39)**	Positive	Cervix (IC)
		9911(34)	Positive	Cervix (IC)
2,2	Control	7819(75)	Negative	Cervix+vagina
	UGC positive	9724(25)	Negative	Cervix (HGIL)
		9770(42)	Negative	Cervix+vagina (LGIL)
3,3	UGC positive	7867(73)	Negative	Cervix (HGIL)
	UGC positive	9339(70)	Negative	Cervix+vagina (HGIL)
2,4	Control	9274(8)	Negative	Cervix

Supplementary table III. Results of tissues other than lower genital tract tissues examined for HPA2 immunolabelling. The only tissue that exhibited immunolabelling was in the stomach of cancer animal 9911(34)

Accession No.	Pv11 genotype	Other tissues	Cancer/Control	Labelling
**9184(14)**	1,1	Urinary bladder<br>Diaphragm	Control	Negative<br>Negative
**9463(28)**	1,1	Ovary<br>Lymph node<br>Heart	Control	Negative<br>Negative<br>Negative
**7972(74)**	1,1	Urinary bladder<br>Heart	Cancer	Negative<br>Negative
**9911(34)**	1,1	Stomach	Cancer	Positive
**7819(75)**	2,2	Lymph node	Control	Negative
**9724(25)**	2,2	Tonsil	Cancer	Negative
**7867(73)**	3,3	Ovary<br>Uterus	Cancer	Negative
**9339(70)**	3,3	Urinary bladder	Cancer	Negative
**9274(8)**	2,4	Ovary<br>Uterus	Control	Negative

Supplementary figures

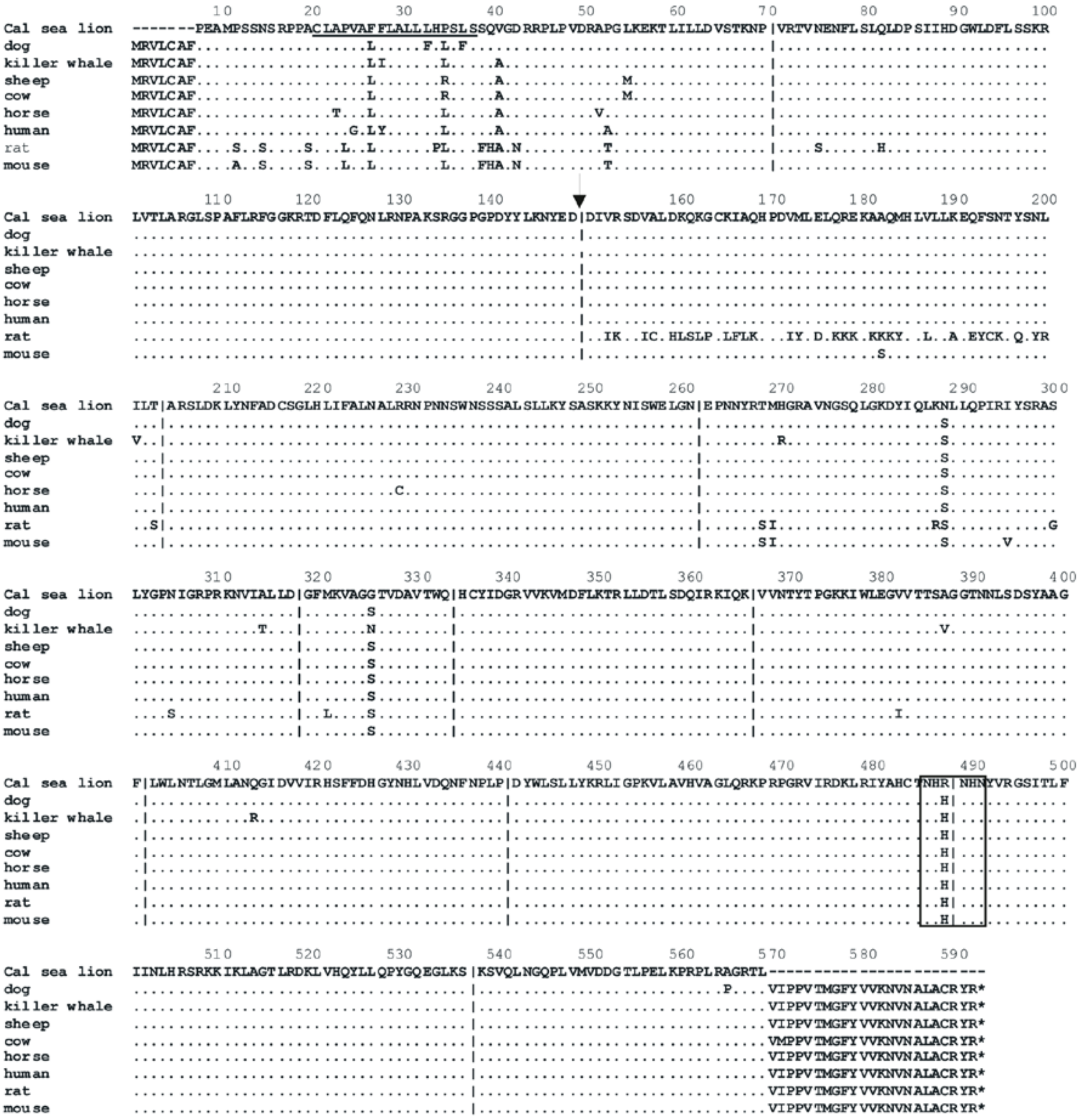

Supplementary figure 1. Amino acid alignment of CSL *HPSE2* predicted protein sequence with representative mammalian species. Dashes represent the regions of CSL *HPSE2* sequence that are not yet known. Identical residues to the CSL are indicated with a '.', vertical lines are the predicted exon/exon boundaries, the underlined sequence is the predicted transmembrane region and the boxed sequence is the predicted heparan binding motif. The exon boundary that the human splice variants arise after is indicated by the arrow.

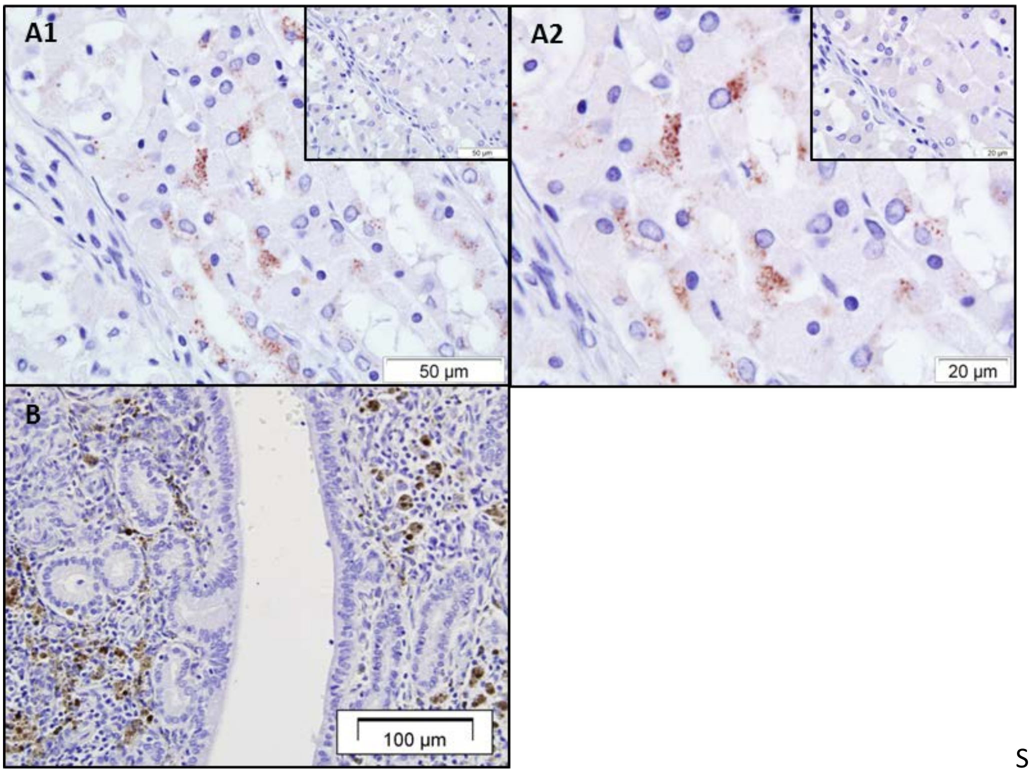

Supplementary Figure 2. Other tissues examined. A1/A2: Animal 9911(34). Positive labelling (shown at x400 and x600) in the stomach with intense granular cytoplasmic labelling within the epithelial cells lining the gastric pits (red pigment) with both peri-nuclear and sub-cell membrane localisation and large areas of the cytoplasm devoid of labelling. Semi-serial negative control sections for each slide are shown inset. B: Animal 7867(73): Uterus (x200) with obvious haemosiderin deposits but no labelling

Supplementary methods

Genotyping – Study B

Following extraction the quantity of gDNA was measured with a Nanodrop spectrophotometer (ND-1000, Thermo Fisher Scientific, USA) and working stocks were made by diluting the samples to 10ng/µl prior to PCR. The three microsatellite markers in the study had previously been amplified in pinnipeds. In addition Pv11 [5] and M11a [6] had been identified in a pilot study as of interest with regards to urogenital carcinoma in California sea lions. Hg8.10 [7] was used as a control. The multiplex PCR consisted of 10µl reactions of 20ng DNA, 5 µl 2×Qiagen Multiplex Master Mix (Qiagen) and 3 µl of primer mix. The primer mix contained 6µM of forward and reverse primer of the three microsatellites (Pv11F: GTG CTG GTG AAT TAG CCC ATT ATA AG, Pv11R: CAG AGT AAG CAC CCA AGG AGC AG; M11aF: TGT TTC CCA GTT TTA CCA, M11aR: TAC ATT CAC AAG GCT CAA; Hg8.10F: AAT TCT GAA GCA GCC CAA G, Hg8.10R: GAA TTC TTT TCT AGC ATA GGT TG) along with 3.3µM of Pv11 forward primer with a D3 fluorescent tag (green), 2.1µM of M11a forward primer with a D4 fluorescent tag (blue) and 3µM of Hg8.10 forward primer with a D2 fluorescent tag (black) and 2.1 µl RNase-free water (Qiagen). Primers were obtained from Invitrogen and fluorescent tags from Sigma. Amplification of the microsatellite markers was carried out in a G-Storm thermo-cycler (G-Storm, UK) using the following temperature cycling conditions; 95°C for 15 min, followed by 35 cycles of 94°C for 30 s, 57°C for 90 s, 72°C for 45 s, followed by a final extension step of 72 °C for 10 min. The samples were stored at 4°C prior to analysis. Before analysis via automated capillary electrophoresis PCR products were diluted with 10µl of milliQ water and 40 µl of a 400bp size standard (Genome lab, Beckman Coulter) was added to each sample. Analysis was then completed using CEQ specific software and the resultant fragments assigned homozygous or heterozygous according to the peaks produced. The sizes of the peaks were recorded and analysed graphically (data not shown) in order to assign alleles to each size group.

Pv11 structure in skin and genital tissue DNA

gDNA extractions samples were quantified as per genotyping. A nested PCR protocol was employed to amplify Pv11 for the purpose of sequencing in order to increase the specificity of the PCR. The reaction mixture for both the primary and nested PCR consisted of 25µl reactions of 2.5µl of *Pfu* DNA polymerase 10X buffer (Promega), 0.5µl 10mM dNTPs (Qiagen), 0.5µl of forward and reverse primer from 50µM stock solutions, 0.21µl *Pfu* DNA polymerase (0.63 units), 19.79µl RNase free water (Qiagen) and 1µl of 10ng/µl DNA template in the case of the primary reaction. For the nested reaction the product of the primary reaction was diluted 1:5 and 1µl of this was used as the template. The primers used were designed based on published sequences of the intron containing Pv11 for the following species; canine, equine, bovine and harbour seal (*Phoca vitulina*). This enabled the identification of conserved regions. The primers sequences are as follows and were obtained from Invitrogen. Primary reaction (a) - Pv11aF: CCT TGA CTT ATC CCT TCA TCT C, Pv11aR: CAG GTG AGG ACC AGG CTC and nested (b) reaction – Pv11bF: CAC CTT TAA CCC ATT GCC TCT G, Pv11bR: CAT TGA GGT GAT GCT GGA AAG. A negative control was included in both the primary and nested reactions. Amplification of the microsatellite marker was carried out in a PTC-0200 DNA Engine Cycler (Bio-Rad Laboratories Inc., USA) using the following temperature cycling conditions for both primary and nested reactions; 95°C for 2 min, followed by 35 cycles of 95°C for 45 s, 50°C for 30 s, 72°C for 4 min, followed by a final extension step of 72°C for 5 min before being stored at 4°C. The expected product size of the nested PCR was approximately 719bp. To analyse the products, 5µl of PCR product was resolved at 80V for 30 min on a 1.5% agarose gel containing 2.5µl ethidium bromide. Bands were visualised in a UV light box (UVITEC, UK). Samples showing successful amplification on gel electrophoresis were purified and then quantified by running 5µl of the purified product alongside three lambda DNA size markers (25ng, 50ng and 100ng) on a 1% agarose gel. Where possible, 40ng of PCR product was submitted for sequencing along with 3.2µM of both forward and reverse primer from the nested (b) reaction.

Loss of Heterozygosity

To investigate potential allele loss, the Pv11 microsatellite marker was amplified alone within skin and corresponding lower genital tract tissue. The Pv11 primer sequences for the PCR are detailed in the genotyping section above. The reactions consisted of 10µl mixtures of 20ng DNA template from either skin or lower genital tract, 5 µl 2×Qiagen Multiplex Master Mix (Qiagen) and 3 µl of primer mix. The primer mix was prepared by combining 6µM of forward and reverse primer along with 3.3µM of Pv11 forward primer with a D3 fluorescent tag (green) and 2.7µl RNase-free water (Qiagen). Primers were obtained from Invitrogen and fluorescent tags from Sigma. Amplification of Pv11 was carried out in a PTC-0200 DNA Engine Cycler (Bio-Rad Laboratories Inc., USA), using the following temperature cycling conditions 95°C for 15 min, followed by 35 cycles of 94°C for 30 s, 57°C for 90 s, 72°C for 45 s, followed by a final extension step of 72 °C for 10 min. The samples were stored at 4°C prior to analysis. Fragment analysis was achieved via automated capillary electrophoresis followed by peak analysis using CEQ specific software as with the genotyping protocol. In addition to ascertaining whether samples where homo or heterozygous, the peak heights of the fluorescent signal were also recorded to enable loss of heterozygosity calculations.

Investigating the location of Pv11 by southern blot

Prior to southern blotting two restriction digests per DNA sample were carried out on 5 ug of genomic DNA using the restriction enzymes BamH1 (New England Biolabs) and HindIII (New England Biolabs) as per manufacturer's guidelines. The DNA was separated on a 1% TBE agarose gel. Two probes were employed one being 1kb in size and flanked the Pv11 microsatellite (probe one) and the second was 800bp in size and incorporated exon 9 of the HPSE2 gene (probe two). To prepare the two probes, PCR's were carried out using California sea lion genomic DNA as template and degenerate PCR primers supplied by Sigma. The sequences of the primers used to make probe A were Forward(Pv11): TGGACCAGAATTTYAACCCA and Reverse(Pv11): CYAAGAGAYGGGTTCCTAC and for probe B Forward(exon): CAGAGRCCYAGAGAATWAATAATCTGCC and Reverse(exon): CAACTGCTCCTTGGGTGCTTACTCTGTG. The 50µl reactions consisted of 10xNH4 buffer (Bioline),

1.25µl 50mM MgCl2, 0.5µl 10mM dNTP (Invitrogen), 1µl of forward and reverse primer from 10µM stock solutions, 0.5µl (2 units) Bio-X-ACT short DNA polymerase (Bioline), 38.75µl of RNase free water and 2µl (124ng/µl) of DNA template. The reaction cycle for probe one was as follows 95°C for 1 min, followed by 35 cycles of 95°C for 1 min, 59°C for 1 min, 72°C for 2 min, then 72°C for 15 min before being stored at 4°C. For probe 2 the same reaction cycle was used with the modification of the annealing temperature being raised to 64°C. The products were resolved on 1% agarose gel and bands of appropriate size extracted and purified using QIAquick gel extraction kit (Qiagen). The probes were then labelled using a DIG-High Prime kit as per manufacturer's instructions (Roche Diagnostics, Germany).

HPSE2 transcription

RNA was extracted from 10-30mg of lower genital tract tissue and quantified with a Nanodrop spectrophotometer (ND-1000, Thermo Fisher Scientific, USA). Following conversion the integrity of the cDNA was achieved by PCRs using primers targeted to the mammalian beta actin gene: BAF: GAGAAGCTGTGCTACGTCGC; BAR: CCAGACAGCACTGTGTTGGC [8]. New England Biolab Taq DNA polymerase with thermopol buffer was used in 25µl reaction mixtures consisting of 2.5µl reaction buffer, 0.5µl 10mM dNTPs (Qiagen), 0.5µl of forward primer and reverse primer from 10µM stock solutions 0.125µl taq (0.625 units) and 18.375µl RNAse free water. 2.5µl of unquantified cDNA was added as template. Amplification was carried out in a PTC-0200 DNA Engine Cycler, (Bio-Rad Laboratories Inc., USA) using the following cycle; 95°C for 30 s followed by 35 cycles of 95°C for 30 s, 53°C for 30 s, 68°C for 60 s. The samples were then held for 5 min at 68°C before being stored at 4°C. Confirmation of successful conversion to cDNA was made by resolving the products on a 1.5% agarose gel with an expected product size of 275bp.

In both the small amplicon and full length amplicon PCR's the primers employed were designed via rapid amplification of cDNA ends (RACE) (Hammond, unpublished/this study). The primer sequences for the small amplicon PCR were: - primary PCR; 105SF: ATGCCCTCCAGCAACTCC, 358ASR:

AATCGAGCCAGCCATCATG and for the nested reaction; 199SF: GAGACAGGAGACCCT TGCC, 358ASR: AATCGAGCCAGCCATCATG. The PCR reaction consisted of 25µl mixtures of 5µl GoTaq® Flexi colourless buffer (Promega), 2.5µl MgCl2 (25mM), 0.5µl 10mM dNTPs (Qiagen), 1.5µl of forward and reverse primer from 10µM stock solutions, 0.125µl GoTaq® Flexi DNA polymerase (0.625 units), 12.875µl RNase free water. 1µl of un-quantified cDNA was used as the template. The primary reaction cycle, carried out in a PTC-0200 DNA Engine Cycler (Bio-Rad Laboratories Inc., USA) was as follows. 94°C for 2 min followed by 35 cycles of 94°C for 20 s, 56°C for 20 s and 72°C for 30 s, the mixtures were then held at 72°C for 7 min before being stored at 4°C. For the nested reaction a 1:5 dilution of the primary PCR product was made and 1µl of this was then used as the template. The reaction mix was the same as the primary mix but the reaction cycle was modified by reducing the cycle number to 30. The presence of HPSE2 isoform was identified by resolving the products of the secondary PCR on a 1.5% agarose gel, the expected product size was 159bp.

To amplify the full length HPSE2 isoform a second hemi-nested PCR was used. Primers for the primary PCR were : HPSE2_5'UTR-S: ATCAGAGGGATTTAATGAGGGTG, HPSE2_3'UTR –AS2: CATGGTGACTGGAGGGATGAC and for the nested reaction; HPSE2_5'UTR-S2: ATGAGGGTGCTCTGTGCCTTC, HPSE2_3'UTR–AS2: CATGGTGACTGGAGGGATGAC. The primary PCR reaction consisted of 25µl reaction mixtures of 2.5µl 10xNH4 buffer (Bioline), 1.25µl 50mM MgCl2, 0.5µl 10mM dNTP (Qiagen), 1µl of forward and reverse primer from 10µM stock solutions, 0.25µl (1 unit) Bio-X-ACT short DNA polymerase (Bioline), 17.5µl RNase free water and 1µl of un-quantified cDNA as template. Amplification was carried out in a PTC-0200 DNA Engine Cycler (Bio-Rad Laboratories Inc., USA) was as follows; 94°C for 2 min, followed by 35 cycles of 94°C for 25 s, 57°C for 20 s, 72°C for 2 min 30 s, before being held at 72°C for 7 min and then stored at 4°C. For the nested reaction a 1:5 dilution of the primary PCR product was made and 2µl of this was used as template DNA. The nested reactions consisted of 50µl reaction mixtures and five reactions for each sample were prepared to give greater yield of product for subsequent cloning. The reactions consisted of; 5µl 10xNH4 buffer (Bioline), 2.5µl 50mM MgCl2, 1µl 10mM dNTP (Qiagen), 2µl of

forward and reverse primer from a 10µM stock solution, 0.5µl (2 units) Bio-X-ACT short DNA polymerase (Bioline) and 35µl RNase free water. The reaction cycle was modified from the primary reaction by reducing the cycle number to 30. The products were resolved at 90V on a 30cm 1.2% agarose gel. The gel was post stained with 15µl of ethidium bromide prior to visualising it in a UV light box (UVITEC, UK) and extracting the bands of correct size for subsequent ligation into pGEM-T easy vector (Promega) and cloning in TOP10 cells (Invitrogen). Following confirmation of successful cloning, plasmid samples with insert of the correct size were quantified against lambda DNA as previously prior to sequencing.

Immunohistochemistry

Sections were dewaxed in xylene and rehydrated through graded alcohols prior to enzymatic antigen retrieval via treating the samples with trypsin. 0.1g of Chymotrypsin (Sigma) was added to 200ml trypsin working solution (20ml 0.1 M $CaCl_2$, 20ml 0.5% trypsin stock solution, 160ml purified water at pH 7.8). The solution was heated to 37°C and the slides incubated for 15 min followed by washing in running tap water (5 min). Blocking endogenous tissue peroxidase activity was then carried out by immersion in 3% H2O2 (v/v) for 20 minutes. Sections were then washed in running tap water (5 min) prior to being immersed in 25% normal rabbit serum (NRS) diluted in phosphate buffered saline (PBS) for 30 minutes at room temperature to block non-specific antibody binding. This was followed by blocking of endogenous tissue biotin binding blocked with a commercial kit (Avidin/Biotin blocking kit, (Vector Laboratories) as per manufacturer's instruction. The primary antibody (polyclonal, goat IgG raised against a peptide of human heparanase 2 (HPA2 (C-17), Santa Cruz Biotechnology, Inc. Ca) was applied diluted 1:100 in 25% NRS/PBS at 4°C overnight. This antibody reportedly cross reacts with a range of species, including the dog. Moreover the epitope is located at the C-terminal end of the protein which is highly conserved in all species. As this region of the protein is highly conserved in CSL *HPSE2* and would be present in all the dominant HPA 2 isoforms based on the cDNA sequencing in this study, we used this antibody to show the cellular

presence and distribution of the protein in the urogenital tract of cancer and control animals of different Pv11 genotypes by immunohistochemistry. A negative control preparation for each of the tissue sections comprised of substituting the primary antibody with normal goat serum at a dilution of 1:100. Slides were rinsed in PBS three times prior to addition of a rabbit anti-goat IgG:biotin conjugate (Vector Laboratories) diluted 1:200 in 25% NRS/PBS for 60 minutes at room temperature. Following a further 3 washes in PBS a commercial ABC avidin biotin kit (Vector Laboratories) was used to amplify the signal from the conjugate as per manufacturer's instructions. The chromogen NovaRED (Vector Laboratories) was applied as per manufacturer's instructions, and the slides were counter stained with haematoxylin, dehydrated, cleared and mounted. The slides were examined by light microscopy (Olympus BX50) and the presence of labelling compared between the UGC positive and UGC negative control samples as well as comparison with the immunohistochemical methodology negative controls. The samples were scored "yes" if clear labelling was present and "no" if labelling was absent. In ambiguous cases findings were noted. There is no recognised standard positive control tissue for HPA2 immunohistochemistry. It is found primarily in neoplastic tissues [1, 2, 9-11] and is therefore is considered its own positive control as long as rigorous negative controls are used in the methodology. Additional tissues (other than those from the lower genital tract) incidentally on the slides were also reviewed for the presence of labelling; these included three sections of urinary bladder and two sections of uterus (supplementary Table III). Previously high mRNA expression has been reported in both urinary bladder and uterus [4, 12] and these tissues were therefore considered as possible positive controls.

References for supplementary material

1. de Moura J.P., Jr., Nicolau S.M., Stavale J.N., da Silva Pinhal M.A., de Matos L.L., Baracat E.C., de Lima G.R. 2009 Heparanase-2 expression in normal ovarian epithelium and in benign and malignant ovarian tumors. *Int J Gynecol Cancer* **19**(9), 1494-1500. (doi:10.1111/IGC.0b013e3181a834a2

00009577-200912000-00006 [pii]).

2. Zhang X., Xu S., Tan Q., Liu L. 2013 High expression of heparanase-2 is an independent prognostic parameter for favorable survival in gastric cancer patients. *Cancer Epidemiol* **37**(6), 1010-1013. (doi:10.1016/j.canep.2013.09.012

S1877-7821(13)00154-9 [pii]).
3. Pang J., Zhang S., Yang P., Hawkins-Lee B., Zhong J., Zhang Y., Ochoa B., Agundez J.A., Voelckel M.A., Fisher R.B., et al. 2010 Loss-of-function mutations in HPSE2 cause the autosomal recessive urofacial syndrome. *Am J Hum Genet* **86**(6), 957-962.
4. McKenzie E., Tyson K., Stamps A., Smith P., Turner P., Barry R., Hircock M., Patel S., Barry E., Stubberfield C., et al. 2000 Cloning and expression profiling of Hpa2, a novel mammalian heparanase family member. *Biochem Biophys Res Commun* **276**(3), 1170-1177. (doi:10.1006/bbrc.2000.3586

S0006-291X(00)93586-1 [pii]).
5. Goodman S.J. 1997 Dinucleotide repeat polymorphisms at seven anonymous microsatellite loci cloned from the European harbour seal (Phoca vitulina vitulina). *Anim Genet* **28**(4), 310-311.
6. Hoelzel A.R., Campagna C., Arnbom T. 2001 Genetic and morphometric differentiation between island and mainland southern elephant seal populations. *Proc Biol Sci* **268**(1464), 325-332. (doi:10.1098/rspb.2000.1375).
7. Allen P.J., Amos W., Pomeroy P.P., Twiss S.D. 1995 Microsatellite variation in grey seals (Halichoerus grypus) shows evidence of genetic differentiation between two British breeding colonies. *Mol Ecol* **4**(6), 653-662.
8. Smolarek-Benson K.A. 2005 Molecular Identification and Genetic Characterization of Cetacean Herpesviruses and Porpoise Morbillivirus, University of Florida.
9. Levy-Adam F., Feld S., Cohen-Kaplan V., Shteingauz A., Gross M., Arvatz G., Naroditsky I., Ilan N., Doweck I., Vlodavsky I. 2010 Heparanase 2 interacts with heparan sulfate with high affinity and inhibits heparanase activity. *J Biol Chem* **285**(36), 28010-28019. (doi:10.1074/jbc.M110.116384

M110.116384 [pii]).
10. Marques R.M., Focchi G.R., Theodoro T.R., Castelo A., Pinhal M.A., Nicolau S.M. 2012 The immunoexpression of heparanase 2 in normal epithelium, intraepithelial, and invasive squamous neoplasia of the cervix. *J Low Genit Tract Dis* **16**(3), 256-262. (doi:10.1097/LGT.0b013e3182422c69).
11. Peretti T., Waisberg J., Mader A.M., de Matos L.L., da Costa R.B., Conceicao G.M., Lopes A.C., Nader H.B., Pinhal M.A. 2008 Heparanase-2, syndecan-1, and extracellular matrix remodeling in colorectal carcinoma. *Eur J Gastroenterol Hepatol* **20**(8), 756-765. (doi:10.1097/MEG.0b013e3282fc2649

00042737-200808000-00008 [pii]).
12. Daly S.B., Urquhart J.E., Hilton E., McKenzie E.A., Kammerer R.A., Lewis M., Kerr B., Stuart H., Donnai D., Long D.A., et al. 2010 Mutations in HPSE2 cause urofacial syndrome. *Am J Hum Genet* **86**(6), 963-969.
